# Acute Suppuration in the Knee-Joint

**Published:** 1909-09-18

**Authors:** 


### Surgery.

### ACUTE SUPPURATION IN THE KNEE-JOINT.

A genuine suppurative arthritis of the knee-joint is one of the most serious conditions that a surgeon can be called upon to deal with; for not only is the subsequent utility of the joint likely to be very seriously impaired or even completely destroyed; but if active surgical interference is not immediately undertaken the life of the patient may be endangered.

Most of the cases are the result of infection from without, caused either by imperfect technique during an operation in which the interior of the joint is exposed (as, for instance, wiring a fractured patella, or the removal of a displaced or loose semilunar cartilage), or by a perforating wound of the joint by a septic instrument. This is due to the fact that the synovial membranes of joints have much less power of resistance than other serous membranes like the peritoneum, which can, and often does, deal with an infection of its own accord. Evidence of this is supplied by those cases of appendicitis which, although moderately acute, often subside spontaneously if nothing is done. Here the peritoneum reacts to the infection, becomes definitely inflamed, and by its own bactericidal power overcomes the infecting agent and removes the toxins, if any have already been formed. This does not occur in the case of synovial membranes. Here the infecting agent runs riot, and, if it be a chæmiotactic organism pus formation occurs and the interior of the joint becomes rapidly disorganised.

For this reason very special care must be taken in preparing the limb, before any operation in which the interior of the joint is to be exposed; and it is well to start three clear days beforehand by shaving the limb and washing it thoroughly with soap and water followed by ether. After this a compress of carbolic acid or biniodide of mercury should be applied and this should be renewed daily up to the time of the operation.

Acute suppurative arthritis of the knee-joint may, however, occur without the introduction of microorganisms from without. In this case the infection is brought by the blood-stream (hæmic) and the process must be regarded as an auto-infective one. Streptococci and staphylococci may make their way into the knee-joint in this way. So also may the pneumococcus; and that without the patient having exhibited any of the ordinary signs of pneumonia. It must be remembered that, although ordinarily speaking a patient infected with the pneumococcus will most often exhibit the disease in its pulmonary form, other organs may be the only ones affected—thus pneumococcal meningitis, peritonitis, arthritis, and even cystitis may occur without any disease of the lungs. Clinically these conditions may be extremely misleading unless this fact is borne in mind. The gonococcus is also capable of producing a suppurative arthritis, but this hardly ever runs so acute a course as the other varieties mentioned.

The first symptom noticed by the patient is pain in the knee, which prevents him first from putting any weight upon it and then from flexing it at all, so that he is forced to take to his bed. Almost at the same time the joint becomes swollen and the outlines of the reflection of the synovial membrane become visible. The pain rapidly becomes excruciating and the temperature rises to 103° F. or higher. The constitutional symptoms are well marked.

On examining the knee, it will be found to be swollen. In the writer's opinion it is easier to detect the presence of fluid by looking at the joint than by palpating it and trying for "the patellar tap." In a typical case a crescentic swelling is seen above the upper border of the patella and a bulging on either side of the ligamentum patellæ. The "patellar tap" is often misleading, not to mention the fact that palpation of the joint is excessively painful. In fact the pain caused by this condition is so great that even accidental movement of the bed is sufficient to make the patient cry out. The surface of the joint is not necessarily red because the pus is beneath the deep fascia.

The only condition which is at all likely to be mistaken for acute suppuration in the joint is a septic prepatellar bursitis; but examination should soon make the differential diagnosis clear. In this the pus is superficial to, and not in the joint; the skin over the bursa is red, and pain is elicited only on palpation of the bursa or on flexing the limb which renders the bursa tense. No pain is caused by jarring the fully extended limb, and the constitutional symptoms are not nearly so severe.

As soon as the diagnosis of suppuration in the joint has been made, no time must be lost in giving a free exit to it. Some surgeons prefer to make an incision on either side of the ligamentum patellæ, and pass an indiarubber tube from one incision to the other after irrigating the synovial cavity. This is sometimes sufficient and the inflammation subsides, if the infection has not been a very virulent one. But in a very acute case sufficient drainage is not obtained thereby. It is then necessary to make a crescentic incision, convex downwards from one side of the joint to the other, cutting through the ligamentum patellæ. The whole interior of the joint can then be exposed by flexing the limb and the cavity can be adequately washed out. Ankylosis often occurs subsequently so that division of the ligamentum patellæ will have done no harm. It should, however, be the aim of the surgeon to prevent ankylosis if possible. The best method of avoiding it is to move the joint daily under gas after it has been opened. Good results are often obtained in this way, and it is claimed that the movement keeps open the stomata of the lymphatics which open on the synovial surface, and that thus the removal of toxins is facilitated. If a movable joint is obtained an attempt will be made subsequently to reunite the divided ligamentum patellæ.

